# A comparative study between transthoracic and transesophageal echo modalities in evaluation of left ventricular deformation

**DOI:** 10.1186/s43044-019-0004-4

**Published:** 2019-08-05

**Authors:** Hala mahfouz Badran, Mahmoud kamel Ahmed, Morad Mena Beshay, Fatma Elzahraa Abdelmonem Zein

**Affiliations:** Menoufia University, 55-ElGish street, P.O Box 34, Tanta, Egypt

**Keywords:** Transoesophageal echocardiography, Left ventricular deformation, 2D strain imaging

## Abstract

**Background:**

2D strain imaging has been proved as an accurate technique for the assessment of left ventricular (LV) function using transthoracic echocardiography (TTE). Transesophageal echocardiography (TEE) has become a standard and essential technique in clinical practice especially when TTE is inconclusive. Adding 2D strain imaging to TEE might be valuable in the evaluation of cardiac performance.

The aim of the present study was to compare 2D strain imaging using TTE and TEE in the assessment of left ventricular deformation in patients with cardiovascular diseases.

**Results:**

This study was conducted on sixty patients, who were referred for TEE for various clinical indications. All patients were examined with both TTE and TEE 2D strain imaging. Global and regional strain parameters including longitudinal (LS), circumferential (CS), and radial (RS) were examined. Analysis of 2D strain using TTE was time sparing than TEE (16 ± 1 vs 19 ± 2 min, *P* < 0.001). From 1020 segments explored using TTE, 97% (LS) and 93% (CS and RS) of the segments were fully analyzed versus 90% and 88% using TEE respectively (*P* < 0.01). TEE longitudinal strain has an excellent agreement with TTE-derived measurements and a modest agreement in circumferential strain but a notable disparity in radial strain values. Both regional and global LS and RS measured by TTE showed higher values (*P* < 0.01, < 0.03) compared with its corresponding values measured by TEE. Whereas segmental and global values of CS were higher using TTE modes, the difference with the analogous TEE values did not reach statistical significance. LS and CS measured by TTE and TEE showed excellent but similar correlation with LV EF and wall motion score index.

**Conclusion:**

2D strain using TTE is user friendly compared to TEE. However, because of the good agreement, TEE 2D strain might represent an appropriate alternative in the evaluation of global LV deformation.

## Background

Transesophageal echocardiography (TEE) has become a very important and efficient technique in clinical practice [1]. It is indicated in any case when the transthoracic examination is inconclusive [2] and in patients who are extremely difficult to be assessed using transthoracic echocardiography (TTE), e.g., postoperative and ventilated patients [3].

Speckle tracking echocardiography (STE) was popularized in the first decade of this century [4]. Analysis of cardiac mechanics has been the focus of ultrasonics, and the breakthrough came with STE. Beyond analysis solely of left ventricular ejection fraction, STE enables an objective assessment of various strain components, longitudinal, circumferential, and radial strain in addition to LV twist, rotation, and dyssynchrony [5, 6].

Transesophageal strain and strain rate measurements were reported to be an effective tool for assessing myocardial deformation [7]. On the other hand, it was found that two-dimensional (2D) strain imaging, a non-Doppler deformation parameter, using TEE 2D strain imaging measurements could be used preoperatively in the examination and evaluation of ventricular function [8]. However, whether TEE strain assessment is superior to TTE 2D strain is not yet clear as evident from the conflicting reports about the level of correlation between TTE and TEE 2D strain.

The objectives of this study were to estimate and compare global longitudinal, circumferential, and radial strains between TTE and TEE modes in patients with cardiac diseases and define its relationship to left ventricular (LV) function using the conventional method.

## Patients and method

This study is a cross-sectional, observational study which included sixty patients who were referred to Menoufia University Hospital for TEE between March 2014 and February 2016. The study protocol was approved by the institutional review board, and patients provided a written informed consent.

*Exclusion criteria*: Patients with one or more of the following were excluded from the study: patients with swallowing problems and history of esophageal disease such as strictures, diverticula, and tumors; patients with recent, gastro-esophageal surgery and high-risk gastro-esophageal bleeding, for example, varices and bleeding ulcer; patients with unsatisfying echocardiographic images, in both TTE and TEE (poor image quality on echocardiography); and patients with homodynamic instability were excluded from the study.

### Methodology

Echocardiography was performed with simultaneous electrocardiography (ECG) recording by a single operator, recordings were performed with subjects in the semi-supine or left lateral positions, and all study population was examined with a phased array 3.5-MHz probe for TTE and a 7.0-MHz probe for TEE.

2D echocardiography assessment included parasternal long- and short-axis views; apical 4-, 3-, and 2-chamber views; and conventional and spectral Doppler imaging. LV, ejection fraction, and LV volumes were calculated using Simpson’s biplane method. Left atrial (LA) size and volume pulsed mitral inflow and tissue Doppler imaging of the lateral and septal mitral annulus were used to assess LV diastolic function.

#### Transesophageal echocardiographic examination (TEE)

All study population was examined with the two modalities in the same setting. After the TTE examination was done, a TEE examination is performed on the same ultrasound system using a TEE probe.

Patients were fasting for at least 4 h; every conscious patient was asked about swallowing problems and any history of esophageal disease; an intravenous line was placed and a supply of oxygen as well as equipment for suction were at hand, especially if sedation is used; and a bite guard was placed. Topical oropharyngeal anesthesia as lidocaine was given.

The probe tip has to be unlocked regarding flexion and extension during intubation of the esophagus. TEE was done with monitoring vital signs of the patients. Mid esophageal views were taken to image LV apical four-, two-, and three-chamber views, and the transgastric examination was done to explore short access of LV at three levels (mitral annulus, papillary muscles, and apical).

The frame rate for both TTE and TEE was between 50 and 90 frames per second, and 3 cardiac cycles were stored in the cine-loop format.

#### 2D strain imaging study

LV apical long-axis views and apical four-, two-, and three-chamber views of both TTE and TEE were acquired, and 3 consecutive cardiac cycles were acquired at end-expiration breath holding and digitally stored on a hard disk for offline analysis. Using 2D strain software, the LV endocardial border was manually traced for both TTE views and TEE views. On the basis of this line, the computer automatically created a region of interest (ROI) including the entire transmural wall, and the software selected suitable natural acoustic markers moving with the tissue for tracking. Image analysis was performed offline on workstation computer using custom analysis software (Echopac PC, version 1.8.1.X, GE Healthcare). Longitudinal strain obtained from apical views four, two, and three of both TTE and TEE and both segmental and global longitudinal strain (LS) strains were calculated. Circumferential and radial strains were assessed from short access views at level of mitral annulus, papillary muscle level, and cardiac apex, and then both segmental and global strains were calculated.

#### Variability study

Inter- and intraobserver variability for strain analysis on TTE images was previously reported with considerable agreement (2.3 ± 0.25% and 2.6 ± 0.25% respectively [6] and 0.4 ± 4.0% and 1.4 ± 4.0% respectively for TTE intraobserver and interobserver variability [7]).

## Statistical analysis

Values are presented as numbers (%) or mean (SD). Unpaired data were analyzed by the chi-square test or unpaired Student test, and paired data were analyzed by the McNemar test or paired Student’s test, as appropriate. The association of continuous data was measured by the Pearson correlation coefficient. Reliability analysis was performed by Cronbach’s alpha, with calculation of intraclass correlation coefficient (95% interval of confidence) and testing its significance with the *F* test. Statistical analysis was performed by SPSS software version 18, all analyses were bilateral, and a *P* value of 0.05 was the limit of statistical significance.

## Results

Demographic and clinical characteristics of the study population are shown in Table 1. The age range was 16–68 years, 19 (32%) patients were males, one (2%) patient was an ex-smoker, 4 (7%) patients were hypertensive, all patients were not diabetic (100%), and the range of BMI was 22–38 kg/m^2^.

**Table 1:** Clinical characteristics, demographic data, and conventional echo variables of our study population

Variable	Patient no. (*N* = 60)	Percent	
Gender			
Male	19	32	
Female	41	68	
Smoking			
Non	59	98	
Ex-smoker	1	2	
HTN	4	6.7	
Nondiabetic	60	100	
Rhythm			
Sinus	50	83.3	
AF	10	16.7	
	Mean ± SD		
Age (years)	40 ± 11	PWD (mm)	9 ± 2
BMI (kg/m^2^)	30 ± 3	LVESD (mm)	33 ± 7
TTE HR (b/min)	81 ± 20	LVEDD (mm)	51 ± 7
TEE HR (b/min)	97 ± 18	FS%	36 ± 6
SBP (mmHg)	117 ± 9	EF%	65 ± 8
DBP (mmHg)	76 ± 5	EDV(ml)	126 ± 45
AO (mm)	30 ± 5	ESV(ml)	46 ± 28
LA (mm)	45 ± 10	WMSI	1 ± 0.2
IVS (mm)	9 ± 2		

*AO* aorta, *LA* left atrium, *IVSD* interventriculer septum in diastole, *PWD* posterior wall in diastole, *LVESD* left ventricular end systolic diameter, *LVEDD* left ventricular end diastolic diameter, *FS* fractional shorting, *EF* ejection fraction, *EDV* end-diastolic volume, *ESV* end-systolic volume, *WMSI* wall motion score index

Fifty (83%) patients were sinus rhythm, and 10 (17%) patients had AF (Table 1); resting heart rate of the studied population was ranged from 56 to131 bpm, while during TEE, it was ranged higher from 62 to 140 bpm, and systolic BP was ranged from 100 to 150 mmHg and the diastolic BP was ranged from 70 to 90 mmHg.

### Indications for TEE

We search for possible intra-cardiac thrombi in patients with cerebral stroke (*n* = 7, 11%); evaluation of atrial septal defect (*n* = 7, 11%); congenital heart disease (*n* = 3, 5%): one had cor-triatriatum, one had bicuspid aortic valve, and one patient had subaortic membrane. Moreover, one patient had aortic valve prolapse, one patient had LA mass, and one had IHD (*n* = 3, 5%). Twenty-six (43%) had rheumatic heart disease [24 had mitral stenosis, one patient had mitral valve prosthesis, and another one had aortic valve prosthesis]. Another one patient had aortic dissection; three patients were referred for comprehensive evaluation before cardioversion. The remaining 10 (16%) patients were referred by an internist for cardiac evaluation.

**Fig. 1:**
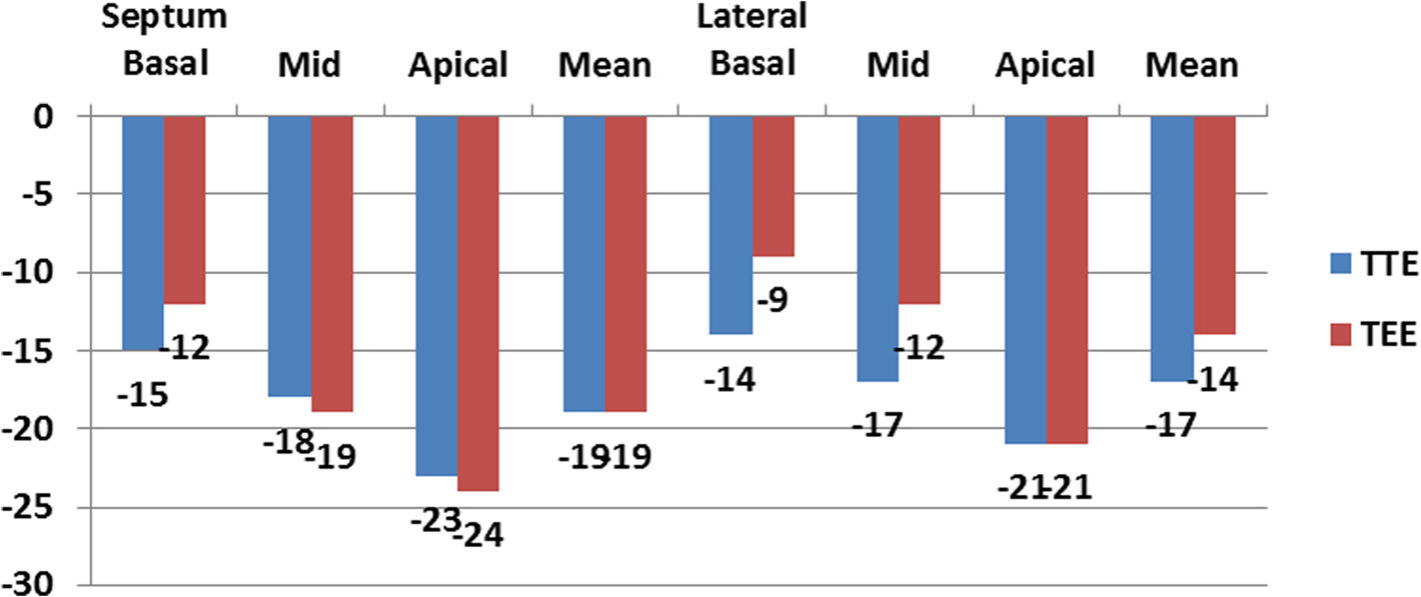
Segmental LV longitudinal strain values using transthoracic versus transesophageal echo (septal, lateral wall segments)

**Fig. 2:**
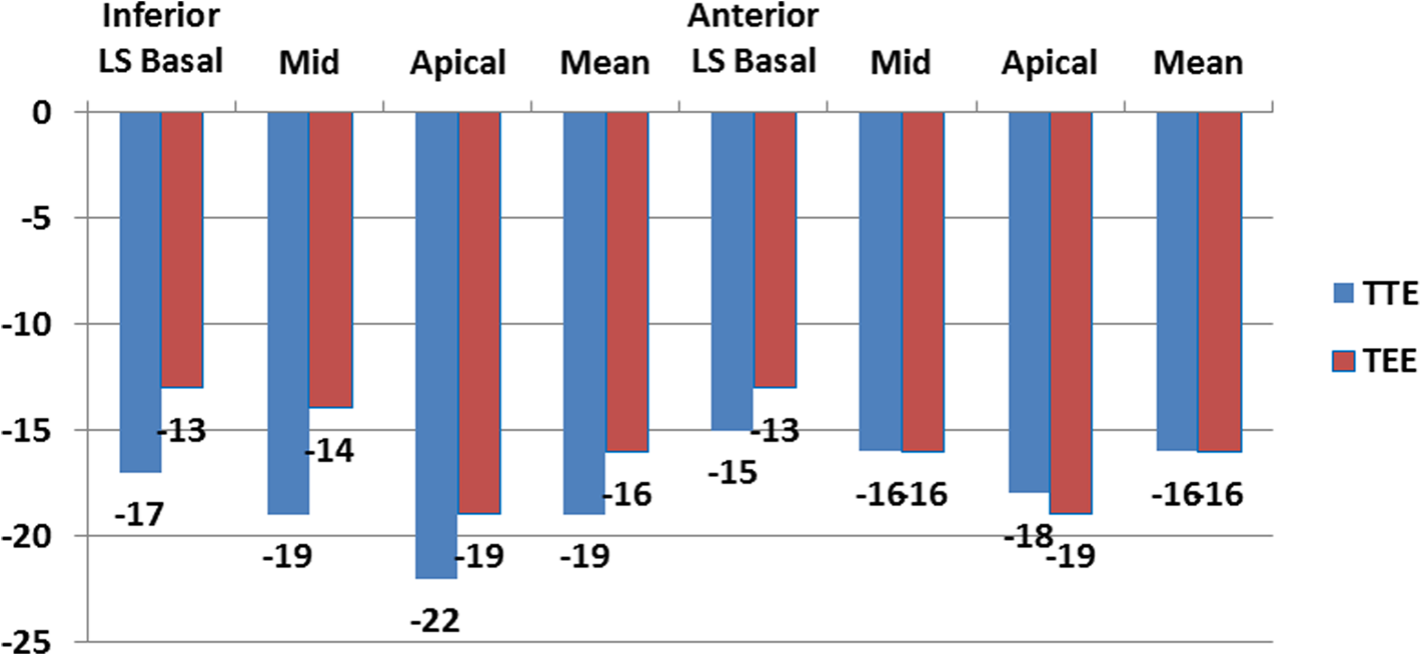
Segmental LV longitudinal strain values using transthoracic versus transesophageal echo (inferior, anterior wall segments)

**Fig. 3:**
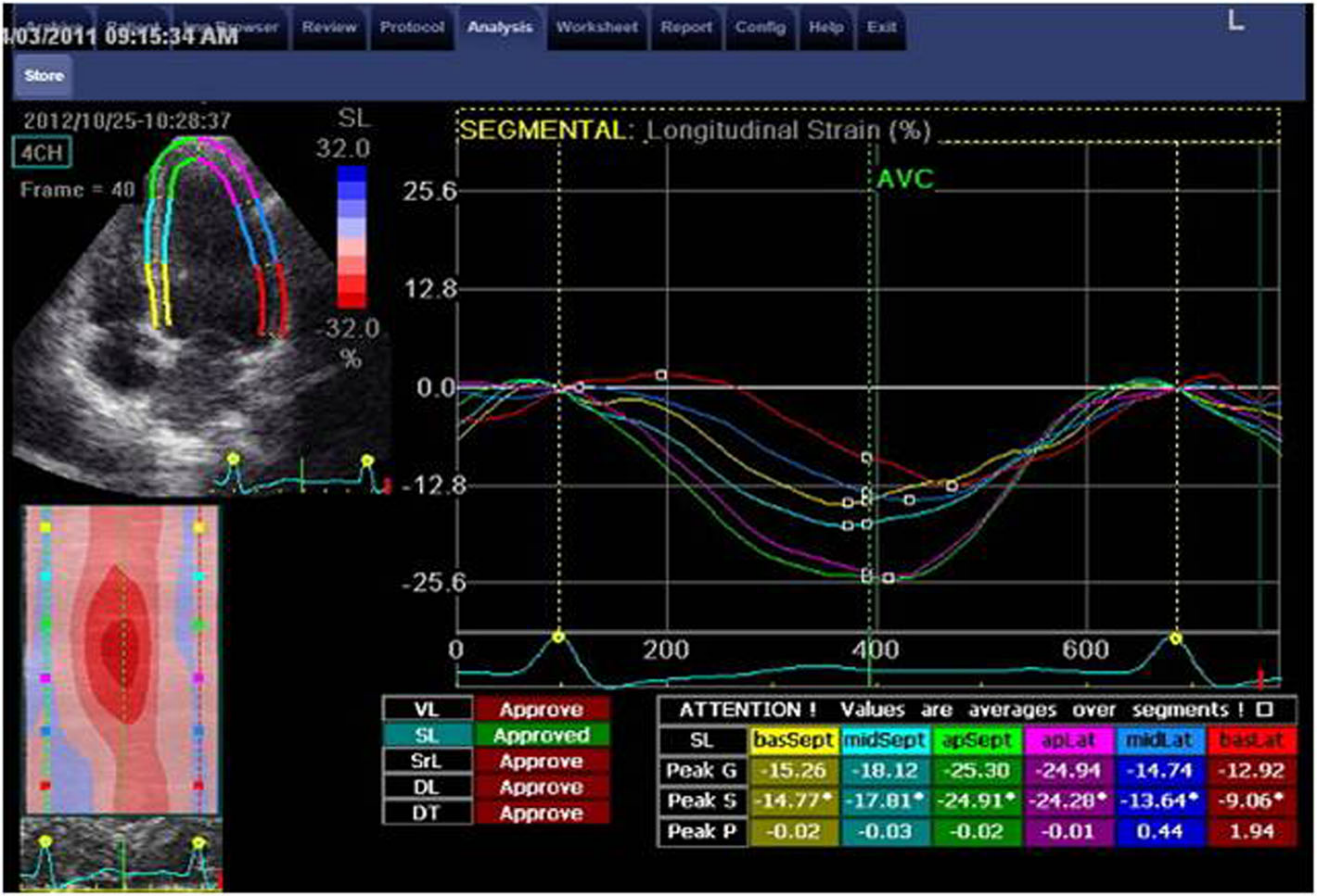
Apical four-chamber view and analysis of septal and lateral wall strain using TTE

**Fig. 4:**
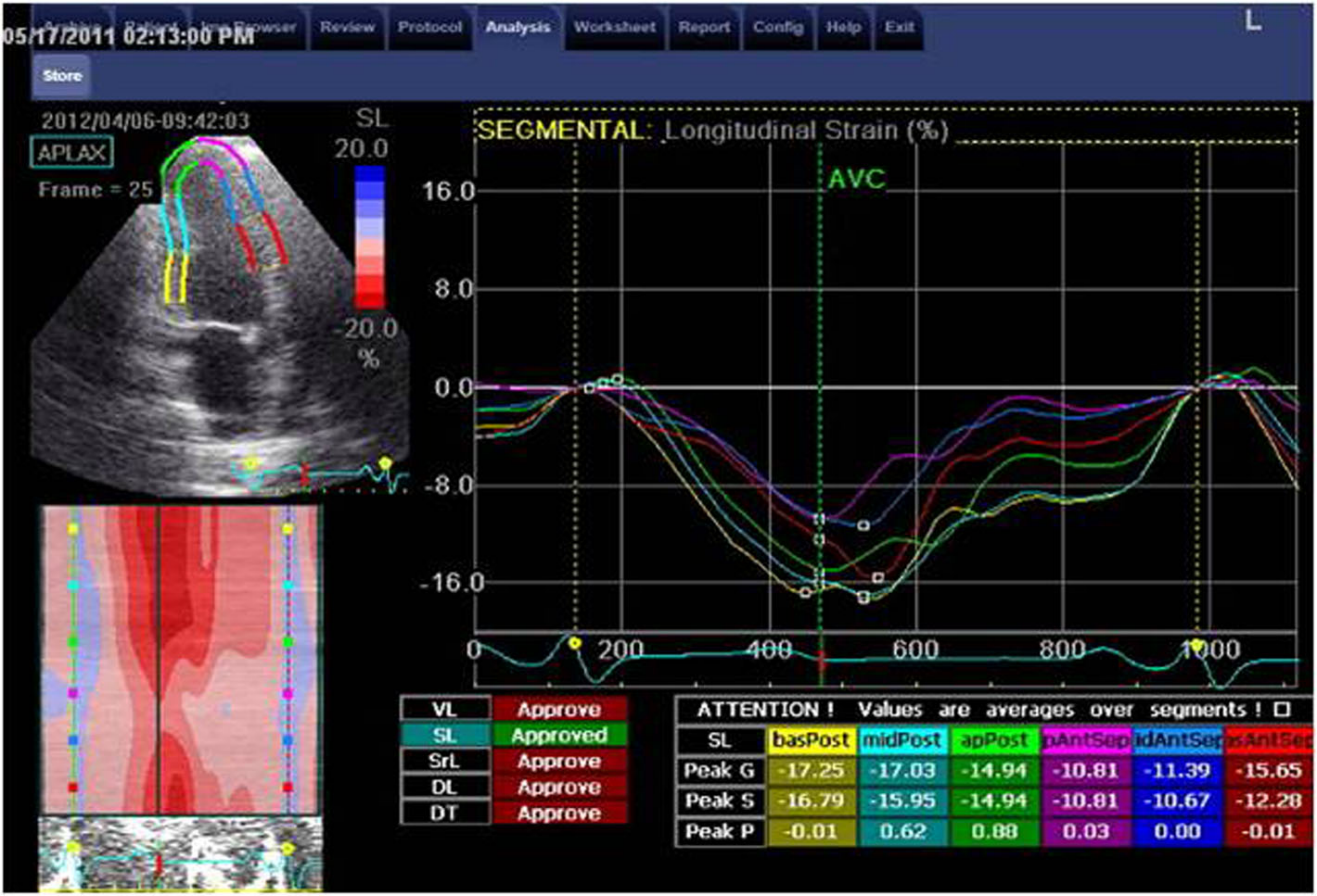
Apical three-chamber view and analysis of posterior and anteroseptal wall strain using TTE

**Fig. 5:**
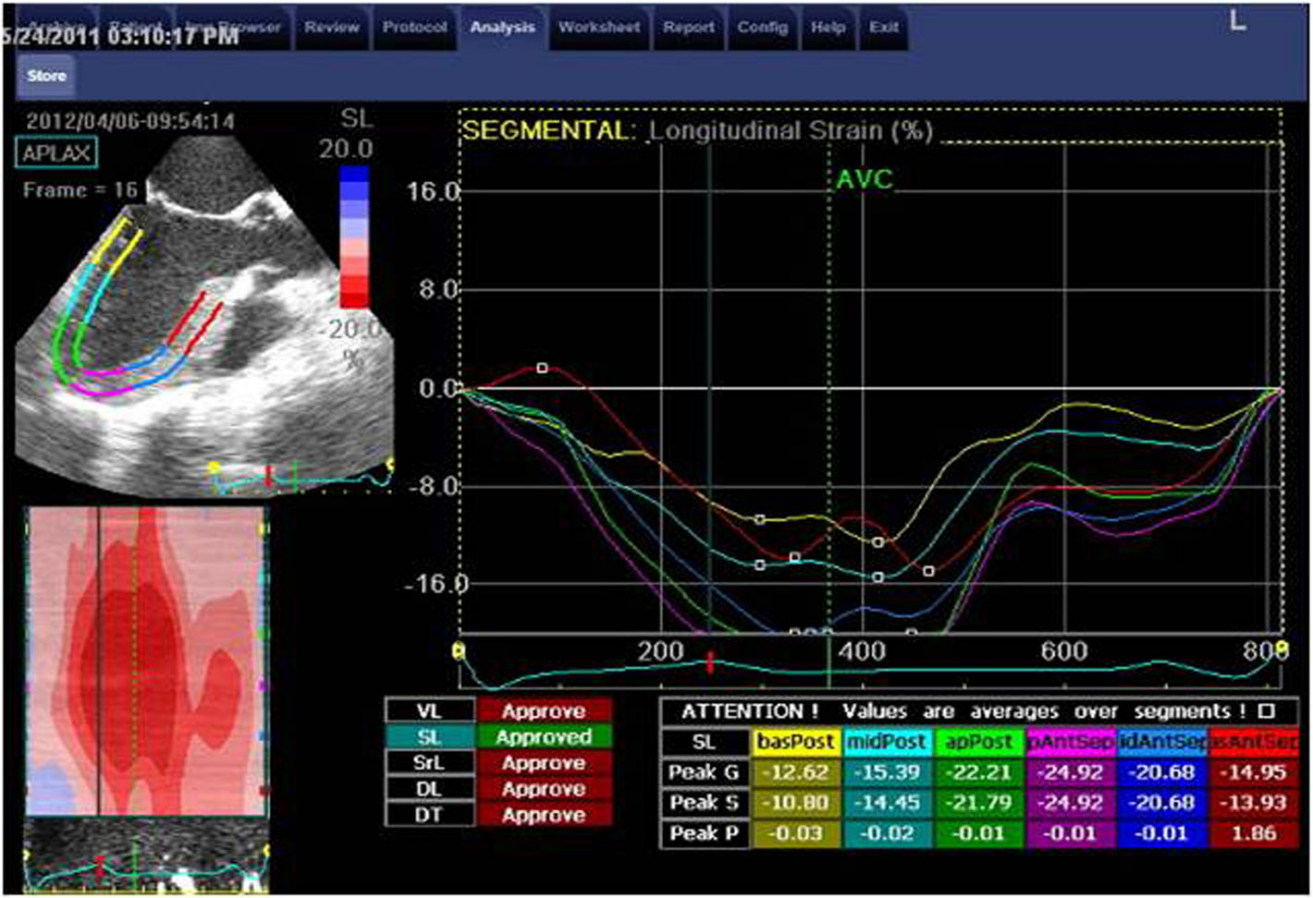
Apical three-chamber view and analysis of posterior and anteroseptal wall strain using TEE

The measurements of longitudinal strain using TTE showed higher values of LS of almost all LV segments in comparison to TEE values. Consequently, the value of GLS was significantly higher when measured by TTE compared to its corresponding value measured by TEE (*P* < 0.05). Only the mid anteroseptal segment showed the reverse, where LS was significantly lower using TTE compared with TEE [− 15 ± 6 vs − 18 ± 7 (*P* < 0.001)] (Figs. 1, 2, 3, 4, and 5; Table 2).

**Table 2:** Comparison between LV longitudinal strain using TTE versus TEE

Segmental Strain (LS) %	TTE Mean ± SD	TEE Mean ± SD	*P* value
Septum			
Basal (%)	− 15 ± 6	− 12 ± 21	0.2
Mid (%)	− 18 ± 6	− 19 ± 9	0.8
Apical (%)	− 23 ± 6	− 24 ± 10	0.2
Mean (%)	− 19 ± 5	− 19 ± 8	0.7
Lateral			
Basal (%)	− 14 ± 6	− 9 ± 9	0.00
Mid (%)	− 17 ± 5	− 12 ± 9	0.00
Apical (%)	− 21 ± 6	− 21 ± 11	0.7
Mean (%)	− 17 ± 5	− 14 ± 8	0.01
Inferior			
Basal (%)	− 17 ± 5	− 13 ± 8	0.00
Mid (%)	− 19 ± 5	− 14 ± 8	0.00
Apical (%)	− 22 ± 5	− 19 ± 9	0.04
Mean (%)	− 19 ± 5	− 16 ± 6	0.00
Anterior			
Basal (%)	− 15 ± 6	− 13 ± 7	0.12
Mid (%)	− 16 ± 6	− 16 ± 7	0.7
Apical (%)	− 18 ± 6	− 19 ± 8	0.24
Mean (%)	− 16 ± 5	− 16 ± 7	1
Anteroseptal			
Basal (%)	− 13 ± 6	− 13 ± 7	0.55
Mid (%)	− 15 ± 6	− 18 ± 7	0.00
Apical (%)	− 17 ± 8	− 19 ± 9	0.054
Mean (%)	− 15 ± 6	− 17 ± 6	0.08
Posterior			
Basal (%)	− 15 ± 6	− 12 ± 8	0.04
Mid (%)	− 16 ± 6	− 13 ± 7	0.00
Apical (%)	− 18 ± 8	− 17 ± 10	0.6
Mean (%)	− 16 ± 6	− 14 ± 7	0.01
GLS (%)	− 17 ± 4	− 16 ± 4	0.01

**Fig. 6:**
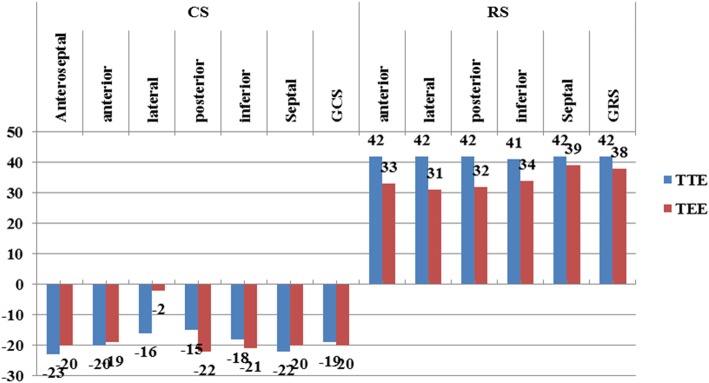
Circumferential and radial strain using TTE and TEE in study groups

Unlike LS, circumferential strain measured at the segmental and global level showed lower values when using TTE compared with TEE. Only CS of the anteroseptal segment was higher using TTE compared to its corresponding value measured by TEE [(− 23 ± 8 vs − 20 ± 10), *P* < 0.05] (Fig. 6). Similar to longitudinal strain, radial strain (RS) measured by TTE showed higher values compared with TEE (Table 3, Fig. 6).

**Table 3:** Comparison between TTE and TEE in circumferential and radial strain

Segmental strain	TTE (CS)	TEE (CS)	*P* value	TTE (RS)	TEE (RS)	*P* value
Anteroseptal (%)	− 23 ± 8	− 20 ± 10	0.03	42 ± 20	33 ± 22	0.01
Anterior (%)	− 20 ± 9	− 19 ± 9	0.9	42 ± 20	31 ± 20	0.00
Lateral (%)	− 16 ± 9	− 0 ± 9	0.00	42 ± 21	32 ± 21	0.00
Posterior (%)	− 15 ± 10	− 22 ± 9	0.00	42 ± 21	34±25	0.03
Inferior (%)	− 18 ± 8	− 21 ± 10	0.03	41 ± 23	39 ± 32	0.6
Septal (%)	− 22 ± 7	− 20 ± 10	0.27	42 ± 20	38 ± 30	0.3
GCS (%)	− 19 ± 7	− 20 ± 8	0.13	42 ± 19	35 ± 23	0.03

*TTE* transthoracic echo, *TEE* transesophageal echo, *GCS* global circumferential strain

In the study population, 1020 segments were explored in longitudinal strain using TTE, 34 segments in 2 patients (3%) were not entirely interpreted, and it includes apical inferior segment in one patient and apical posterior segment in other patients, while using TEE 102 segments 6 patients (10%) were not completely interpreted. It included basal lateral in 2 patients, both basal lateral and basal posterior wall in 2 patients, mid inferior in one patient, and basal septal in another one.

On assessment of both LV circumferential and radial strain by TTE, which is obtained from short-axis view at the level of mitral valve, papillary muscle level, and apical levels, 68 segments in 4 patients (7%) were not feasible on analysis; it includes lateral and posterior walls in one patient and all segments in the remaining 6 patients. Meanwhile, during circumferential strain and radial strain in TEE, 119 segments in 7 patients (12%) could not be analyzed.

**Fig. 7:**
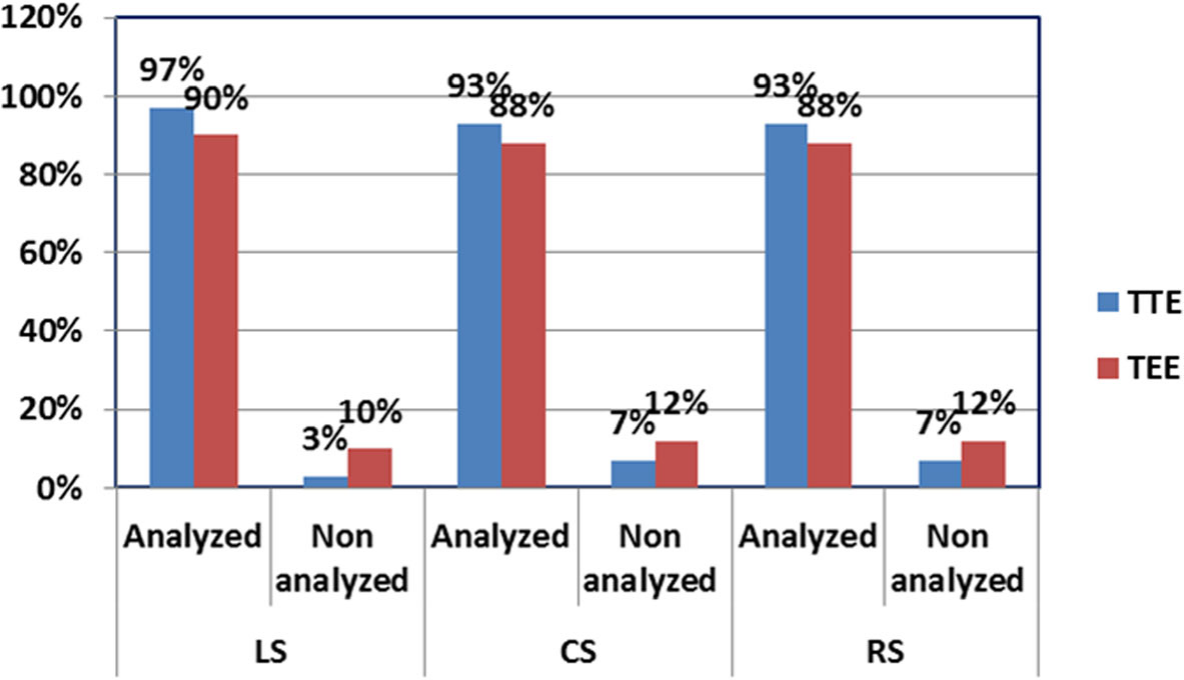
Comparison between TTE and TEE in segmental feasibility for analysis

TTE analysis was more feasible in all strain components: longitudinal, circumferential, and radial strain in comparison to TEE examination (Fig. 7).

TTE acquisition time (7 ± 0.7 vs 10 ± 0. 8 vs 7 ± 0.7 min, *P* < 0.0001), analysis time (7 ± 0.7 vs 10 ± 0.7 min, *P* < 0.001), and total time (14 ± 1 vs 19 ± 1 min, *P* < 0.0001) were significantly shorter in comparison to TEE, which concludes that TEE was more time consuming (*P* < 0.001).

Correlation of LV deformation with conventional ECHO parameters using TTE showed a negative correlation between global LV strain with almost all conventional ECHO parameters, unlike fractional shortening and ejection fraction which were directly correlated with both global LS (*P* < 0.001) and global CS (*P* < 0.05) (Table 4).

**Table 4:** Relation of LV deformation using TTE and TEE to conventional ECHO parameters

ECHO parameters		TTE LS	TTE CS	TTE RS		TEE LS	TEE CS	TEE RS
AO	*r*	− 0.2	0.04	− 0.2	*r*	− 0.2	− 0.1	0.1
	*P*	0.08	0.7	0.1	*P*	0.06	0.5	0.7
LA	*r*	− 0.5	− 0.2	− 0.3	*r*	− 0.5	− 0.4	− 0.3
	*P*	0.00	0.1	0.03	*P*	0.000	0.001	0.03
IVSD	*r*	− 0.02	0.1	0.2	*r*	− 0.1	− 0.07	− 0.02
	*P*	0.9	0.3	0.2	*P*	0.4	0.6	0.9
PWD	*r*	− 0.3	− 0.3	− 0.09	*r*	− 0.2	− 0.3	− 0.07
	*P*	0.05	0.01	0.5	*P*	0.1	0.02	0.6
LVESD	*r*	− 0.4	− 0.3	− 0.2	*r*	− 0.4	− 0.3	0.1
	*P*	0.002	0.04	0.2	*P*	0.001	0.04	0.6
LVEDD	*r*	− 0.2	− 0.2	− 0.2	*r*	− 0.3	− 0.2	0.1
	*P*	0.06	0.08	0.2	*P*	0.01	0.1	0.3
FS%	*r*	0.5	0.3	0.1	*r*	0.4	0.3	0.07
	*P*	0.000	0.03	0.4	*P*	0.000	0.02	0.6
EF%	*r*	0.5	0.3	0.1	*r*	0.5	0.3	0.06
	*P*	0.000	0.03	0.3	*P*	0.000	0.02	0.6
EDV	*r*	− 0.2	− 0.2	− 0.1	*r*	− 0.3	− 0.2	0.1
	*P*	0.06	0.1	0.3	*P*	0.01	0.1	0.4
ESV	*r*	− 0.3	− 0.26	− .147	*r*	− 0.4	− 0.3	0.07
	*P*	0.004	0.052	0.275	*P*	0.001	0.07	0.6
WMSI	*r*	− 0.5	− 0.2	− 0.3	*r*	− 0.5	− 0.3	− 0.1
	*P*	0.000	0.1	0.05	*P*	0.000	0.02	0.4

*AO* aorta, *LA* left atrium, *IVSD* interventricular septum in diastole, *PWD* posterior wall in diastole, *LVESD* left ventricular end-systolic diameter, *LVEDD* left ventricular end-diastolic diameter, *FS* fractional shorting, *EF* ejection fraction, *EDV* end-diastolic volume, *ESV* end-systolic volume, *WMSI* wall motion score index

Consequently, a negative correlation was observed between global LV strain with almost all conventional ECHO parameters using TEE, unlike fractional shortening and ejection fraction which were directly correlated to global LS and CS (*P* < 0.001, < 0.05).

**Fig. 8:**
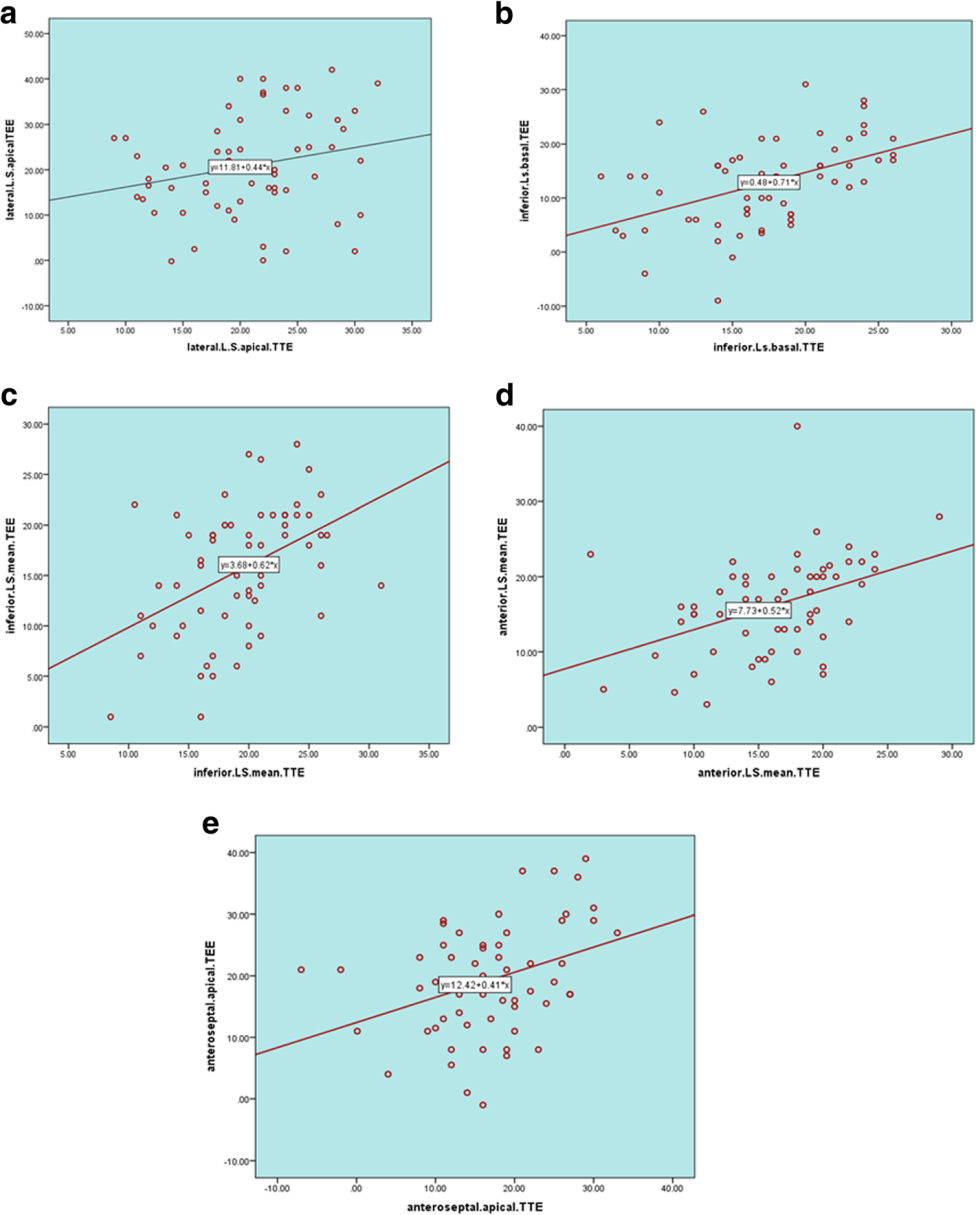
**a**–**e** Correlation of LV segmental longitudinal strain measured by TTE versus TEE in the studied population. **a** Relation of TTE to TEE values of apical lateral LS. **b** Relation of TTE to TEE values of basal inferior LS. **c** Relation of TTE to TEE values of mean inferior wall LS. **d** Relation of TTE to TEE values of mean anterior wall LS. **e** Relation of TTE to TEE values of anteroseptal wall LS. Relation of TTE to TEE values of apical posterior LS

**Fig. 9:**
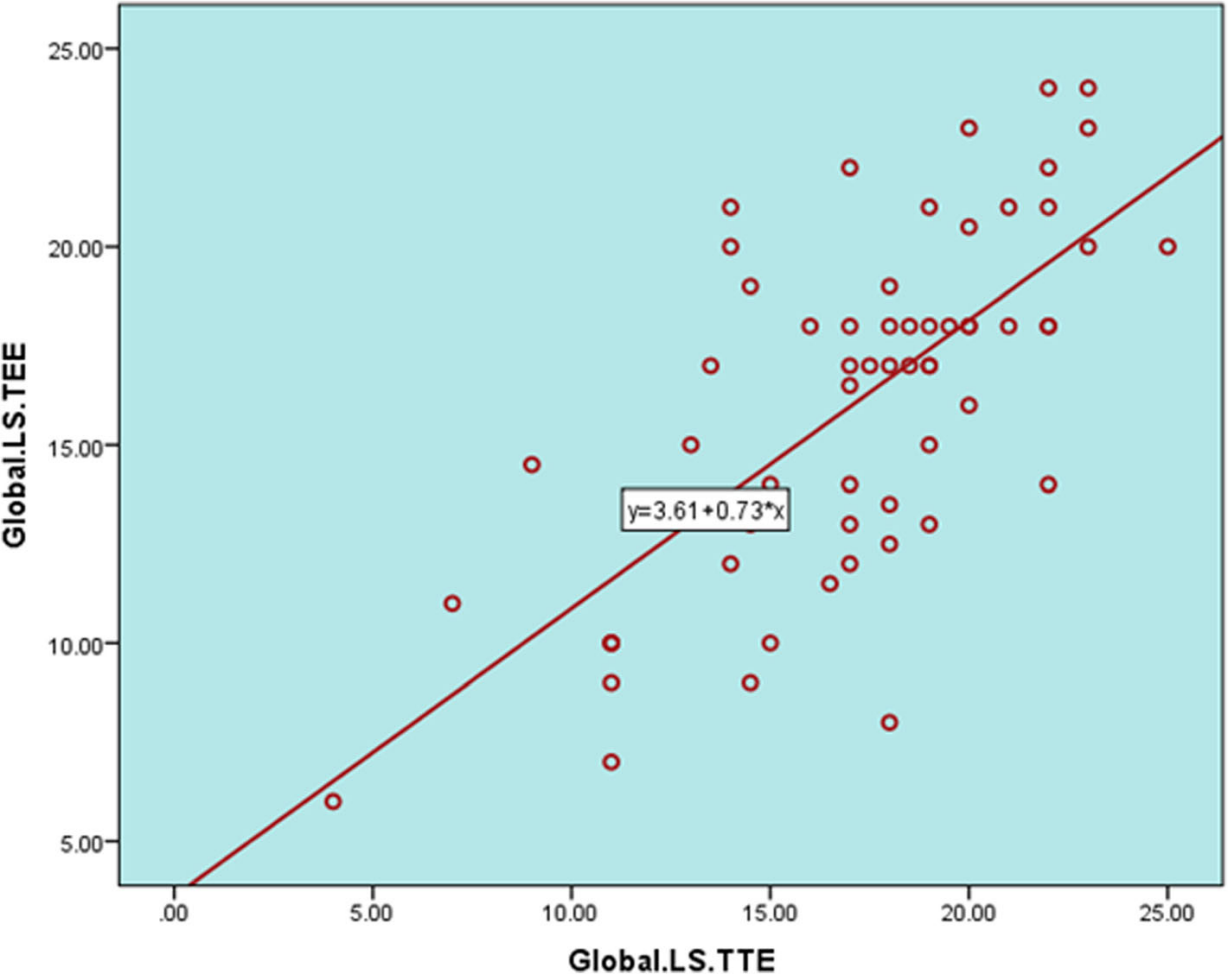
Relationship of LV global longitudinal strain measured by TTE versus TEE in the studied population (*r* = 0.70, *P* < 0.00001)

**Fig. 10:**
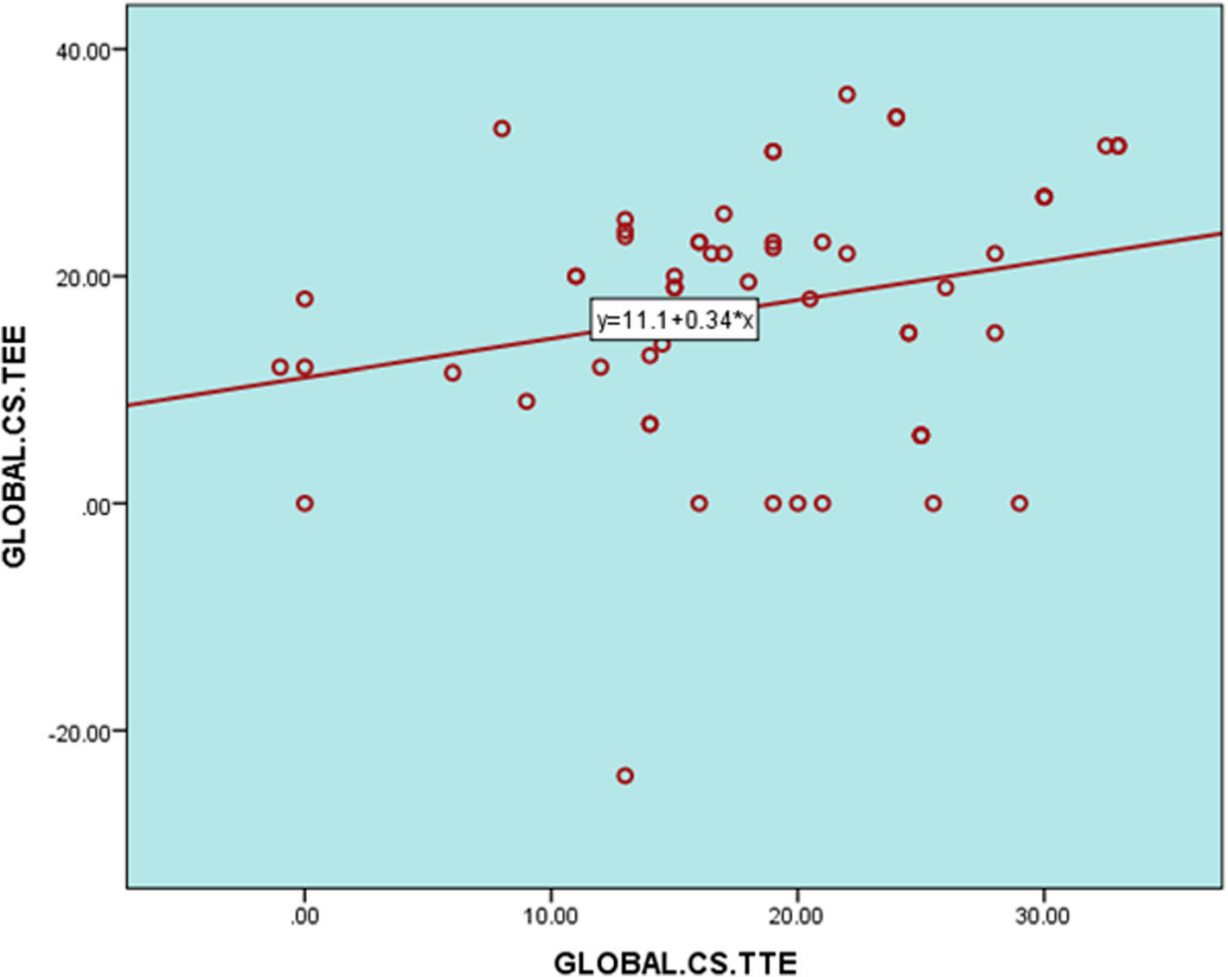
Correlation of LV global circumferential strain as measured by TTE versus TEE in the studied population (*r* = 0.30, *P* < 0.02)

Correlation of LV longitudinal strain segmental values using TTE versus TEE showed a positive correlation. A strong direct relationship was observed between global LS results using TTE and TEE (*P* < 0.001) (Figs. 8 and 9 and Table 5)*.* Similar to LS, CS measurements of both methods showed a positive correlation (Fig. 10, Table 5).

**Table 5:** Agreement of LV longitudinal strain-, circumferential strain-, and radial strain-derived values between TTE and TEE

Segmental strain	*r*	*P* value
Septum		
Basal	0.1	0.4
Mid	0.3	0.02
Apical	0.4	0.001
Mean	0.4	0.001
Lateral LS		
Basal	0.05	0.7
Mid	0.1	0.6
Apical	0.001	0.9
Mean	0.04	0.7
Inferior LS		
Basal	0.5	0.000
Mid	0.193	0.1
Apical	0.2	0.1
Mean	0.4	0.000
Anterior LS		
Basal	0.3	0.03
Mid	0.3	0.007
Apical	0.2	0.03
Mean	0.4	0.001
GLS	0.7	0.000
GRS	*0.2*	*0.2*
Anteroseptal LS		
Basal	0.3	0.02
Mid	0.4	0.003
Apical	0.4	0.005
Mean	0.4	0.002
Posterior LS		
Basal	0.2	0.07
Mid	0.2	0.07
Apical	0.3	0.02
Mean	0.3	0.03
CS		
Anteroseptal	0.4	0.005
Anterior	0.2	0.1
Lateral	0.2	0.08
Posterior	0.01	0.9
Inferior	−0.02	0.9
Septal	0.5	0.000
RS		
Anteroseptal	0.2	0.2
Anterior	0.2	0.2
Lateral	0.2	0.1
Posterior	0.1	0.3
Inferior	0.07	0.6
Septal	0.1	0.3
GCS	*0.3*	*0.02*

## Discussion

We demonstrated that TTE global longitudinal and radial strain values were higher and circumferential strain values were lower than their corresponding values using TEE. TEE longitudinal strain has an excellent agreement with TTE-derived measurements and a modest agreement in circumferential strain but a notable disparity in radial strain values. 2D strain imaging using TTE was more feasible and less time consuming compared with TEE 2D strain measurements. Both modalities were correlated with LV function parameters using conventional echocardiography.

Strain imaging is defined as myocardial deformation imaging, a technique that helps the calculation of both left and right ventricle function. A growing body of evidence shows that the assessment of myocardial strain provides great value in the clinical setting [9], so guidelines now recommend assessment of strain values during the routine assessment of ventricular function [10].

Two modalities have been developed to assess ventricular deformation or strain. Tissue Doppler imaging suffers from the limitations of Doppler technique, most notably angle dependence. The second method, speckle tracking echocardiography, measures strain with acoustic markers or “speckles” on B-mode imaging and tracks their motion relative to one another. Although this method requires an adequate frame rate, it has emerged as a more robust method of strain measurement because speckles can be tracked at any angle [11, 12].

In recent years, this technique of strain imaging progressively became more used in the assessment of global ventricular function [11–15]. As it is reproducible, and able to provide quantitative data, the application of 2D strain imaging in the evaluation of ventricular function is further increased. Transthoracic 2D strain imaging has been studied extensively; there are a great number of articles in the literature describing its clinical application^.^[13]; however, there are a limited number of studies that investigate the relevance of 2D strain imaging during TEE examination [16, 17].

TEE provides important clinical information in the emergency room and patients undergoing cardiac surgery as well as in noncardiac surgery. TEE is used widely often to assess the systolic and diastolic LV functions in the intraoperative and perioperative period [18, 19]. Moreover, TEE is an established imaging modality for patients with inadequate transthoracic acoustic windows in terms of assessing the LV function and management of high-risk patients. That is why, the agreement between TEE and TTE in LV deformation assessment might be an important addition in the echocardiographic laboratory [20–25].

The present study is somewhat different from other TEE 2D strain imaging studies. Kurt et al. [8] studied the reproducibility of TEE 2D strain imaging parameters in 34 healthy individuals with mean age 36 ± 9.2 years, in the absence of any structural cardiovascular disease. The authors concluded that there were generally good agreements between strain and strain rate measurements on TEE and TTE. The inter- and intraobserver agreement for TEE parameters was good.

In the present study, we investigated 2D strain imaging using both TTE and TEE in different cardiac pathologies including valvular heart disease, IHD, cardiomyopathy, congenital heart disease, and stroke which is a real-life daily practice. The age group was close to Kurt et al.’s study age group (16–68 years), and the mean age was 39 ± 11 years. All components of LV strain were investigated longitudinal, circumferential, and radial strain. There was an excellent agreement of longitudinal strain using both TTE and TEE and a modest agreement in circumferential strain and diverting values in radial strain.

In another study, Kukucka et al. [26] speculated that strain calculation from TEE images was feasible. Their study also included patients undergoing CABG surgery, and only TEE measurements were studied.

Tousignant et al. [27] performed a study in which 21 patients underwent CABG surgery, and TTE and TEE values were obtained just for the right ventricle. In this study, the global right ventricular strain value was similar using both methods (20.1% vs 20.4%).

In the present study, TEE 2D strain imaging for the assessment of ventricular function was time consuming and less feasible, when compared with TTE 2D strain, because of the requirement of offline analysis with the workstation. The presence of automated functioning imaging program available in Vivid-9 echocardiographic machines, which is the computer-based program that proposed to give similar data about the global and 4CH, long-axis, and 2CH strain imaging in a short time, failed to shorten the analysis time in addition to longer acquisition time as invasive procedure in comparison to TTE 2D strain analysis [28, 29].

In the current study, the authors found notable differences in mean longitudinal strain and radial strain values of all LV segments between the TTE and TEE measurements (higher in TTE LS and RS and lower in CS). This is likely caused by different technology used in transthoracic and transesophageal modalities in obtaining the best transgastric view for measurements of circumferential and radial strain.

Moreover, our study results indicate that both TTE and TEE LS were highly correlated to LV dimension, volumes, and systolic function indices and WMSI assessed by conventional echocardiographic methods; however, the agreement was weaker in TTE- and TEE-derived CS. TTE and TEE RS were incompatible with all these quantitative parameters’ assessment of LV systolic function. Ryczek et al.’s [30] results were in accordance with our study and demonstrated a strong significant linear correlation between TTE GLS and LVEF.

Aksakal et al. [31] investigated the agreement between TTE and TEE in the assessment of LV systolic functions by longitudinal myocardial deformation imaging (strain-S and strain rate-Sr) and LV diastolic functions by conventional Doppler parameters. The authors demonstrated that both TTE and TEE were correlated to diastolic function parameters assessed by conventional echo-Doppler parameters.

In accordance with the present study, Simmons et al. [32] reported good agreement on Doppler-derived strain and strain rate when comparing intraoperative TEE with TTE. In the present study, the number of segments fully analyzed by TTE in all strain components longitudinal, circumferential, and radial was notably higher than that explored by TEE examination (97% vs 90% in longitudinal strain and 93% vs 88% in radial and circumferential analysis).

In comparison with our study, Carlo et al. showed poor agreement between segmental strain measurements assessed by TTE and TEE regarding longitudinal, circumferential, and radial strain; in the long-axis views, only 193 of the 342 segments that were tracked in both TTE and TEE were used to assess agreement on hypokinetic segments. For LS, 78 segments were graded as normal by both techniques, and 23 segments were scored as normal in TTE but hypokinetic in TEE. Sixty segments were found to be hypokinetic by both techniques, and 32 segments graded hypokinetic by TTE were scored as normal by TEE.

Regarding radial strain in their study, TTE and TEE agreed on only 38 segments graded as normal. Forty-nine segments were normal in TTE and hypokinetic in TEE. Sixty-two segments were hypokinetic for both, whereas 44 segments were hypokinetic only for TTE.

In accordance with our results, Cheung et al. [33] showed that in the animal study during incremental atrial pacing data of TTE and TEE were comparable. In this study, they evaluated peak isovolumic velocity, isovolumic acceleration during isovolumic contraction, and diastolic E and A velocities from data obtained through TTE and TEE-TDI. Using isovolumic acceleration and isovolumic velocity from TTE-TDI, they found that systolic function evaluation had values comparable to those of TEE. However, they concluded that a velocity was incomparably different. Relying on these results, they have reported that TEE-TDI might be suitable for the monitorization of serial changes in LV function.

### Study limitations

Our study was carried out on a limited number of subjects. 2D strain imaging is dependent on good 2D image quality. For this reason, poor 2D image quality results in a poor success rate. LV deformation assessment was only for systolic function using speckle tracking, and diastolic function was not analyzed with strain imaging.

Finally, although normal values for TTE strain and strain rate in a general population have been described, “normal” values for healthy individuals using TEE imaging are not available.

## Conclusion

With the improvement in TEE technologies that we have seen since its introduction, it is expected that further improvement and introduction of the novel imaging modalities will continue to be introduced in the coming years. In this study, application of TEE strain in the evaluation of LV function in varying cardiac diseases was compared to that of TTE. Strain analysis using both modalities, TTE and TEE, is comparable. However, TTE strain was more feasible and more correlated to conventional echo variables used for LV function assessment like ejection fraction and RWMA. TEE longitudinal strain has an excellent agreement with TTE-derived measurements and a modest agreement in circumferential strain but a notable disparity in radial strain values. While TTE 2D strain-derived values were more feasible and less time consuming compared with TEE 2D strain measurements, both modalities were correlated to conventional ECHO parameters used for LV function assessment.

## Data Availability

The dataset supporting the results and conclusions of this article will be available from the corresponding author on request.

## Abbreviations

2D: Two dimensional
ECG: Electrocardiography
LA: Left atrial
LS: Longitudinal strain
LV: Left ventricular
STE: Speckle tracking echocardiography
TEE: Transesophageal echocardiography
TTE: Transthoracic echocardiography
